# Effect of protected fatty acid supplementation on ovarian activity, reproductive hormone profiles and reproduction of Barki ewes under semiarid conditions

**DOI:** 10.5194/aab-67-111-2024

**Published:** 2024-03-14

**Authors:** Bahaa Farrag, Khalid Ahmed El-Bahrawy, Hesham Attia Shedeed, Muhammed Ahmed-Hilmy El-Rayes

**Affiliations:** Animal Physiology Department, Animal and Poultry Production Division, Desert Research Center (DRC), Cairo, Egypt

## Abstract

The objective of this experiment was to evaluate the impact of dietary supplementation with rumen-protected fat (calcium soaps of fatty acids, CSFA) and/or hormonal superovulation using equine chorionic gonadotropin (eCG) on ovarian activity, changes in ovarian hormones, and reproductive performance in Barki ewes under semiarid conditions. A total of 39 non-pregnant Barki ewes, with an average body weight of 34.7 ± 0.25 kg and an average age of 3–4 years, were divided into four groups. The first group (9 ewes) served as the control, while the second group (10 ewes) received double doses of PGF ${}_{2}\alpha$ , administered 9 d apart, and was superovulated with 600 IU (international unit) eCG. The third group (10 ewes) was supplemented with 30 g per head per day of rumen-protected fat. The fourth group (10 ewes) underwent the same superovulation protocol as the second group and was also supplemented with 30 g per head per day of rumen-protected fat. All the ewes were fed a basal diet. Ovarian activity was detected using ultrasonography during the estrus phase in all the groups, after which all the ewes were naturally bred with Barki rams. The results demonstrated significant ( P<0.05 ) increases in estradiol 17- β (E ${}_{2}$ ) and progesterone (P ${}_{4}$ ) levels in the treated groups compared to the control group. Ewes in all the treated groups exhibited large follicles, with an overall mean diameter ( ≥5 mm) significantly higher than that of ewes in the control group. Although there were no significant differences between the treated and control groups, the ewes treated with CSFA and/or the eCG hormone showed tendencies to improve the reproductive traits of Barki ewes. Overall, the findings of this study demonstrated a positive effect of CSFA alone or in combination with an estrous synchronization protocol on ovarian activity in Barki ewes, as indicated by the number and size of follicles and the presence of large-sized follicles.

## 1 Introduction

Fertility is a crucial factor in sheep productivity, and the number of offspring per lambing serves as a reliable indicator (Petrović, 2000). Under subtropical conditions, the breeding season coincides with summer, characterized by high temperatures, humidity, and a scarcity of high-quality forage (Hashem and El-Zarkouny, 2014). Various experiments have been conducted on ewes to enhance their productive and reproductive performance. These include improving nutrient intake before mating season (Scaramuzzi and Radford, 1983), administering exogenous gonadotropins (Motlomelo et al., 2002), or supplementing with fat, such as calcium soaps of fatty acids (CSFA), which have been shown to affect the reproductive performance of Barki ewes (Hegazy et al., 1999). However, due to limited information regarding the optimal level and appropriate type of fat, extensive research is required in sheep nutrition. Furthermore, implementing dietary measures designed to increase reproductive efficiency may serve as a viable alternative to costly hormonal therapies (Martin et al., 2004).

Early studies by Burr and Burr (1930) examining the effect of fat in the diet on reproductive performance observed that fat deficiency in the diet of growing rats led to alterations in ovulation rate and the onset of estrus. However, reproductive performance was restored upon lipid supplementation, introducing the concept of essential fatty acids. This is particularly relevant as ruminants hydrogenate polyunsaturated fatty acids (PUFAs) in the rumen, limiting the absorption of PUFAs from the small intestine (Thatcher and Staples, 2007; Santos et al., 2008; Doreau et al., 2011). Nevertheless, certain specific PUFAs may bypass ruminal hydrogenation and be absorbed directly from the small intestine. This allows for potential improvements in reproductive efficiency by directly affecting the target tissues of the female reproductive system (autocrine or paracrine) or through indirect effects mediated by the endocrine system (Staples and Thatcher, 2005).

Energy intake represents a significant factor influencing reproductive activity and appears to be the primary determinant of reproductive performance in sheep, compensating for any nutritional deficiencies that may arise. Additionally, in ruminants, a diet rich in fat can increase caloric density without reducing fiber intake, leading to increased energy consumption and utilization efficiency (Espinoza et al., 1998). Supplemental lipids have been employed to enhance the energy density of the diet and overcome negative associative effects often experienced with cereal grains (Coppock and Wilks, 1991; Bowman and Sanson, 1996) while still maintaining favorable rumen fermentation in cows (Chalupa et al., 1986). Fat supplementation, especially with CSFA, is an important technique that can potentially improve productivity by directly or indirectly affecting reproductive function in various important tissues (Funston, 2004).

Early studies demonstrated that supplementing Ghezel ewes' diet with 40 g of protected fat per day increased the number of ovarian follicles larger than 7 mm in diameter (Zamiri et al., 2002) and elevated the circulating progesterone concentration in cyclic ewes supplemented with CSFA (Liel et al., 2010). Furthermore, Hegazy et al. (1999) noted that pregnancy, fertility, and prolificacy rates were higher in Barki ewes fed a diet supplemented with CSFA compared to the control group. Ewes supplemented with long-chain fatty acids in the form of Ca-soap palm oil exhibited improvements in the numbers and sizes of preovulatory follicles, ovulation rates (El-Shahat and Abo-El Maaty, 2010), and conception and lambing rates (Hashem and El-Zarkouny, 2014). Supplementing the diet with fat also led to increased serum and follicular fluid cholesterol, serum progesterone levels, lifespans of induced corpus luteum (CL), the number of medium-sized follicles, and growth of preovulatory follicles (Williams and Stanko, 2000).

The level of blood progesterone is a crucial factor in maintaining pregnancy. Previous studies have indicated that a physiological mechanism is responsible for increasing the progesterone concentration in the serum by reducing the removal rate of progesterone from the blood or potentially increasing the concentration of its precursor (cholesterol) (Hawkins et al., 1995).

On the other hand, various hormones have been administered to enhance ewe fertility and achieve two lambings per year or three lambings in 2 years (Bazer et al., 2007); increase the number of pregnant females in a flock (Motlomelo et al., 2002; Husein et al., 2005); and improve conception rates and fertility, particularly under semiarid conditions (Husein and Kridli, 2003). Employing estrus synchronization techniques has also proven to be effective in inducing estrus in a large number of females simultaneously (Panhwar, 2007).

Therefore, this study aims to evaluate the impact of dietary supplementation with rumen-protected fat (CSFA) and/or superovulation through hormonal treatment (equine chorionic gonadotropin – eCG) on ovarian activity, changes in ovarian hormones, and the reproductive activity of Barki sheep in semiarid environments.

## 2 Materials and methods

### 2.1 Animals and management

This study used 39 non-pregnant Barki ewes with an average body weight of 34.7 ± 0.25 kg and an average age of 3–4 years. The study was conducted at Ras-Sudr Research Station, which belongs to the Desert Research Center, Egypt, located in the South Sinai Governorate of Egypt (29°62 ${}^{\prime }$ N, 32°71 ${}^{\prime }$ E). The ewes were housed in semi-open shaded pens throughout the experimental period. They were fed a commercial concentrate feed mixture based on their body weights and physiological status (NRC, 1985). Additionally, Egyptian clover hay (*Trifolium alexandrinum*) was provided as a roughage ration ad libitum. Fresh water was made available twice daily. The ewes underwent clinical examinations and were found to be free from any diseases or reproductive disorders. The chemical composition of the diets is presented in Table 1. All the experimental procedures were conducted in accordance with the European Union (EU) directive for the protection of experimental animals (2010/63/EU).

**Table 1:** Ingredients and nutrient contents of basal diet rations; diets were formulated in agreement with the nutrient requirements of NRC (1985) for sheep.

Chemical composition	g kg ${}^{-1}$	Ingredients of the concentrate mixture	%
Organic matter	940	Yellow corn	25
Ash	60	Cottonseed meal	16.7
Crude protein	148	Wheat bran	30
Ether extract	55	Sunflower meal	25
Neutral detergent fiber	534	NaCl	1
Acid detergent fiber	369	Limestone	2
Hemicellulose	165	Trace minerals	0.3

### 2.2 Diet regimens and experimental design

The ewes were randomly divided into four groups. The control group (G1, n=9 ) received a basal diet. The second group (G2, n=10 ) received double doses (250 µ g) of cloprostenol acetate (FG ${}_{2}\alpha$ ), 9 d apart, and were superovulated by a single dose (600 IU) of eCG. The third group (G3, n=10 ) was supplemented with 30 g per head per day of rumen-protected fat (Magnapac^®^). Finally, the fourth group (G4, n=10 ) received double doses (250 µ g) of cloprostenol acetate, 9 d apart, and was superovulated by a single dose (600 IU) of eCG in addition to supplementation with 30 g per head per day of rumen-protected fat. The fat added (Magnapac^®^) consisted of CSFA and contained 84 % fat and 9.9 % Ca, with 36.9 % palmitic acid, 33.6 % oleic acid, 7.9 % linoleic acid, 4.2 % stearic acid, and 1.2 % myristic acid. The chemical composition of CSFA is presented in Table 2 according to Younis et al. (2012). The feeding diets were administered for 45 d, with a 10 d acclimatization period before estrus synchronization, and continued for 35 d during estrus synchronization and neutral mating.

**Table 2:** Chemical composition (g kg ${}^{-1}$ ) of the protected fat (calcium soaps; Magnapac^®^).

Item	g kg ${}^{-1}$
Total fat	840.0
Myristic acid	12.6
Palmitic acid	369.6
Stearic acid	42.0
Oleic acid	336.0
Linoleic acid	79.8
Ash	110.0
Ca ${}^{++}$	9.9
Moisture	50.0

### 2.3 Estrus synchronization and induction of synchronized multiple ovulations

Females in groups G2 and G4 received double intramuscular doses (250 µ g) of cloprostenol acetate (Estrumate, Schering-Plough Animal Health, Germany), 125 µ g each and 9 d apart, and were superovulated by a single intramuscular dose (600 IU) of eCG (Folligon^®^, Intervet Corp., Canada).

#### 2.3.1 Monitoring ovarian activity

**Figure 1:**
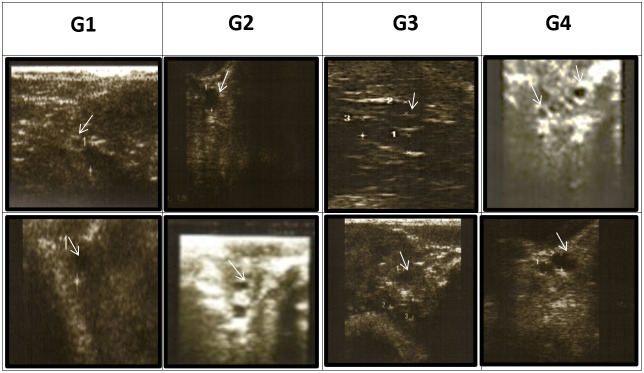
Effect of treatment of CSFA supplementation and/or superovulation with hormonal treatment on the activity of the ovaries of Barki ewes by using ultrasonography. Numerous follicles are between 3 and 5 mm in diameter. (A) First-type diameter of small follicles ( <3 mm), (B) second-type diameter of medium follicles (3–5 mm), and (C) third-type diameter of large follicles ( ≥5 mm) according to Hashem et al. (2015).

Transrectal ultrasonography of the uterus was performed using a 5.0–7.5 MHz linear-array transducer connected to a portable B-mode ultrasound scanner (Eickemeyer Magic 5000 Digital) at 24 ( $T_{24}$ ), 48 ( $T_{48}$ ), and 72 ( $T_{72}$ ) h during the synchronization period. The number, diameter, and relative position of all the ovarian antral follicles were recorded and categorized according to diameter as follows: small follicles (DsmF) with a diameter of less than 3 mm, medium follicles (DMeF) with a diameter of 3–5 mm, and large follicles (DLaF) with a diameter of 5 mm or greater, according to Hashem et al. (2015) (Fig. 1).

#### 2.3.2 Mating and early pregnancy diagnosis

Ewes that exhibited estrus were naturally mated using mature fertile rams. Ultrasound scanning was performed 30 d after mating to examine uterine contents.

### 2.4 Blood samples collected

Blood samples were collected in the morning before access to feed from the jugular vein using Vacutainer tubes (K3 EDTA, 5 mL) for the determination of estradiol 17- β (E ${}_{2}$ ) concentrations at 0 ( $T_{0}$ ), 24 ( $T_{24}$ ), 48 ( $T_{48}$ ), and 72 ( $T_{72}$ ) h and progesterone (P ${}_{4}$ ) concentrations on days 8 (luteal stage), 18, and 30 (early pregnancy) after mating. The plasma was harvested after centrifugation at 5000 g for 10 min and stored at -20 °C for later analysis.

#### Hormonal assay

E ${}_{2}$ and P ${}_{4}$ levels were analyzed using an ELISA kit (Monobind, USA) following the method described by Abraham (1974). The intra- and inter-assay coefficients of variation (CVs) were 9.3 % and 9.7 %, respectively. The optical densities for the hormonal assay were measured using a Stat Fax 2000 ELISA reader.

### 2.5 Statistical analysis

The data were analyzed using the MIXED (mixed-model) procedure for repeated measurements in SAS (2006) according to the following statistical model:

1 $$Y_{ijk}=\mu +T_{i}+M_{j}+TM_{ij}+e_{ijk},$$

where $Y_{ijk}$ is the analyzed trait, μ is the overall mean, $T_{i}$ is the effect of time, $M_{j}$ is the effect of the treatment, TM ${}_{ij}$ is the interaction between the time and treatment, and $e_{ijk}$ is the error effect.

Fixed effects of treatment, time, and their interactions were analyzed for follicle-related variables and serum progesterone and estradiol concentrations. Variables measured only once per animal were analyzed using the GLM (general linear model) procedure of SAS, with treatment as the main effect in the model. Effects of time and its interaction with treatment were displayed only when statistically significant or approaching significance. Categorical data or data expressed as percentages (conception rate, prolificacy rate, and lambing rate) were analyzed using a chi-squared test. Data collected only once, such as the number of CLs, diameters of CLs, and P ${}_{4}$ concentrations, were analyzed using t tests. All the results were expressed as means ± standard errors. Statistical significance was accepted at P<0.05 .

## 3 Results

The data presented in Table 3 revealed no significant differences in the overall mean diameter of small follicles ( <3 mm) among the treated groups (G1, G2, G3, and G4). The respective mean diameters were 2.19 ± 0.09, 2.11 ± 0.09, 2.37 ± 0.09, and 2.42 ± 0.09 mm. Similarly, there were no significant differences in the overall mean diameters of mid-sized ovarian follicles (3–5 mm) among the ewes in groups G1, G2, G3, and G4, with respective mean diameters of 3.86 ± 0.10, 3.89 ± 0.1, 3.97 ± 0.10, and 3.86 ± 0.10 mm. However, a significant difference was observed in the overall mean diameters of large-sized ovarian follicles ( ≥5 mm) in ewes from the treated groups (G2, G3, and G4), with respective mean diameters of 5.55 ± 0.11, 5.40 ± 0.11, and 5.89 ± 0.11 mm, compared to ewes in the control group (G1) with a mean diameter of 5.03 ± 0.11 mm. Among the treated groups, ewes fed CSFA and superovulated with eCG (G4) exhibited the largest-sized ovarian follicles.

**Table 3:** Effect of CSFA and/or superovulation with hormonal treatment on ovarian activity (diameter and number follicles) at different times after mating.

Follicle characteristics	Time (h)	Groups				Overall time	P value		
		G1	G2	G3	G4		G	T	G × T
SMFD Small follicles <3 mm in diameter	24	2.10 ± 0.18	1.97 ± 0.18	2.32 ± 0.18	2.45 ± 0.18	2.21 ± 0.08	0.10	0.61	0.96
	48	2.26 ± 0.18	2.27 ± 0.18	2.37 ± 0.18	2.40 ± 0.18	2.32 ± 0.08			
	72	2.22 ± 0.18	2.10 ± 0.18	2.42 ± 0.18	2.42 ± 0.18	2.29 ± 0.08			
	Overall group	2.19 ± 0.09	2.11 ± 0.09	2.37 ± 0.09	2.42 ± 0.09				
MSFD Mid-sized follicles (3–5 mm)	24	3.30 ± 0.18 ${}^{c}$	3.40 ± 0.18b ${}^{c}$	4.17 ± 0.18 ${}^{a}$	3.97 ± 0.18 ${}^{\mathrm{ab}}$	3.71 ± 0.09	0.98	0.10	0.05
	48	4.10 ± 0.18 ${}^{a}$	4.10 ± 0.18 ${}^{a}$	3.90 ± 0.18 ${}^{\mathrm{abc}}$	3.75 ± 0.18 ${}^{\mathrm{abc}}$	3.96 ± 0.09			
	72	4.20 ± 0.18 ${}^{a}$	4.17 ± 0.18 ${}^{a}$	3.66 ± 0.18 ${}^{\mathrm{abc}}$	3.87 ± 0.18 ${}^{\mathrm{abc}}$	3.97 ± 0.09			
	Overall group	3.86 ± 0.10	3.89 ± 0.10	3.97 ± 0.10	3.86 ± 0.10				
LGFD Mature follicles ( ≥ 5 mm)	24	5.03 ± 0.22 ${}^{b}$	5.43 ± 0.22 ${}^{\mathrm{ab}}$	5.92 ± 0.22 ${}^{a}$	5.60 ± 0.22 ${}^{\mathrm{ab}}$	5.49 ± 0.10	0.01	0.51	0.01
	48	5.07 ± 0.22 ${}^{b}$	5.25 ± 0.22 ${}^{b}$	5.12 ± 0.22 ${}^{b}$	6.06 ± 0.22 ${}^{a}$	5.37 ± 0.10			
	72	5.00 ± 0.22 ${}^{b}$	5.97 ± 0.22 ${}^{a}$	5.15 ± 0.22 ${}^{b}$	5.12 ± 0.22 ${}^{b}$	5.53 ± 0.10			
	Overall group	5.03 ± 0.11 ${}^{C}$	5.55 ± 0.11 ${}^{B}$	5.40 ± 0.11 ${}^{B}$	5.89 ± 0.11 ${}^{A}$				
No. of SMFs	24	1.50 ± 0.38 ${}^{\mathrm{bc}}$	1.25 ± 0.38 ${}^{c}$	2.00 ± 0.38 ${}^{\mathrm{bc}}$	2.75 ± 0.38 ${}^{b}$	1.87 ± 0.19	0.05	0.21	0.05
	48	1.66 ± 0.38 ${}^{\mathrm{bc}}$	1.25 ± 0.38 ${}^{c}$	2.00 ± 0.38 ${}^{\mathrm{bc}}$	4.00 ± 0.38 ${}^{a}$	2.22 ± 0.19			
	72	1.25 ± 0.38 ${}^{c}$	2.66 ± 0.38 ${}^{b}$	1.50 ± 0.38 ${}^{\mathrm{bc}}$	1.50 ± 0.38 ${}^{\mathrm{bc}}$	1.72 ± 0.19			
	Overall group	1.47 ± 0.23 ${}^{B}$	1.72 ± 0.23 ${}^{B}$	1.50 ± 0.23 ${}^{B}$	2.75 ± 0.23 ${}^{A}$				
No. of MEFs	24	1.25 ± 0.49	1.25 ± 0.49	2.75 ± 0.49	1.25 ± 0.49	1.62 ± 0.25 ${}^{B}$	0.26	0.01	0.19
	48	2.25 ± 0.49	3.00 ± 0.49	2.33 ± 0.49	3.50 ± 0.49	2.77 ± 0.25 ${}^{A}$			
	72	2.00 ± 0.49	2.25 ± 0.49	3.00 ± 0.49	2.33 ± 0.49	2.25 ± 0.25 ${}^{\mathrm{AB}}$			
	Overall group	1.83 ± 0.28	2.16 ± 0.28	2.69 ± 0.28	2.77 ± 0.28				
No. of LGFs	24	1.00 ± 0.42 ${}^{c}$	2.33 ± 0.42 ${}^{\mathrm{ab}}$	2.75 ± 0.42 ${}^{a}$	1.33 ± 0.42 ${}^{\mathrm{bc}}$	1.85 ± 0.18	0.14	0.25	0.05
	48	1.75 ± 0.42 ${}^{\mathrm{abc}}$	1.50 ± 0.42 ${}^{\mathrm{abc}}$	1.25 ± 0.42 ${}^{\mathrm{bc}}$	2.66 ± 0.42 ${}^{a}$	1.79 ± 0.18			
	72	1.00 ± 0.42 ${}^{c}$	2.00 ± 0.42 ${}^{\mathrm{abc}}$	1.25 ± 0.42 ${}^{\mathrm{bc}}$	1.50 ± 0.42 ${}^{\mathrm{abc}}$	1.43 ± 0.18			
	Overall group	1.25 ± 0.22	1.94 ± 0.22	1.75 ± 0.22	1.83 ± 0.22				

${}^{a,b,c}$ Values within rows with different superscripts differ significantly ( P<0.05 ). ${}^{A,B,C}$ Means in the same column and row not followed by the same superscript letter are significantly different ( p<0.05 ). G1: control group. G2 received double doses (250 µ g) of cloprostenol acetate and 9 d apart and was superovulated by a single dose (600 IU) of PMSG. G3 was supplemented with 30 g per head per day of rumen-protected fat (Magnapac^®^). G4 received double doses (250 µ g) of cloprostenol acetate and 9 d apart and was superovulated by a single dose (600 IU) of PMSG, in addition to being supplemented with 30 g per head per day of rumen-protected fat. SMFD: small follicles <3 mm in diameter. MSFD: mid-sized follicles (3–5 mm). LGFD: mature follicles ( ≥5 mm). No. of SMFs: number of small follicles. No. of MEFs: number of mid-sized follicles. No. of LGFs: number of large-sized follicles.

Regarding the number of small-, mid-, and large-sized follicles, the results showed that the overall mean number of small follicles ( <3 mm in diameter) was significantly higher (2.75 ± 0.23 mm) in ewes treated with a combination of CSFA and superovulation (G4) compared to the other groups (G1, G2, and G3) with respective mean numbers of 1.47 ± 0.23, 1.72 ± 0.23, and 1.50 ± 0.23. However, there was no significant enhancement in the number of mid-sized or large-sized follicles among all the treated and control ewes (Table 3).

The concentration of estradiol 17- β (E ${}_{2}$ ) was affected ( P<0.05 ) among the groups (Table 4). The results revealed that the overall mean estradiol 17- β concentration was higher ( P<0.05 ) in G4 (57.45 ± 1.34 pg mL ${}^{-1}$ ), followed by G2 and G3 (52.83 ± 1.34 and 51.96 ± 1.34 pg mL ${}^{-1}$ ), respectively, compared to the control group (33.21 ± 1.34 pg mL ${}^{-1}$ ).

**Table 4:** Variations of blood plasma estradiol 17- β (E ${}_{2}$ ) concentrations (pg mL ${}^{-1}$ ) at four times of the synchronization period (0, 24, 48, and 72 h) in the experimental groups.

Experimental group	Estradiol 17- β profile				± SE	Overall	± SE
	Time of synchronization (h)						
	0 ( $T_{0}$ )	24 ( $T_{24}$ )	48 ( $T_{48}$ )	72 ( $T_{72}$ )			
G1	28.14 ${}^{g}$	35.51 ${}^{\mathrm{fg}}$	40.48 ${}^{\mathrm{ef}}$	28.72 ${}^{g}$	2.69	33.21 ${}^{C}$	1.34
G2	37.90 ${}^{f}$	55.91 ${}^{\mathrm{bcd}}$	58.37 ${}^{\mathrm{bc}}$	59.16 ${}^{\mathrm{abc}}$		52.83 ${}^{B}$	
G3	51.22 ${}^{\mathrm{cd}}$	53.91 ${}^{\mathrm{bcd}}$	61.80 ${}^{\mathrm{ab}}$	41.33 ${}^{\mathrm{ef}}$		51.96 ${}^{B}$	
G4	56.30 ${}^{\mathrm{bcd}}$	58.15 ${}^{\mathrm{bc}}$	67.30 ${}^{a}$	48.06 ${}^{\mathrm{de}}$		57.45 ${}^{A}$	
Overall	43.34 ${}^{C}$	50.77 ${}^{B}$	56.99 ${}^{A}$	44.32 ${}^{C}$	1.34		

SE: standard error. ${}^{\text{a–g}}$ : means in the same rows not followed by the same superscript letter are significantly different ( p<0.01 ). ${}^{A,B}$ : means in the same column not followed by the same superscript letter are significantly different ( p<0.05 ).

Estradiol levels were affected ( p≤0.01 ) throughout the synchronization period ( $T_{0}$ – $T_{72}$ h). The estradiol 17- β concentration increased ( P<0.05 ) from $T_{0}$ and gradually reached high levels prior to ovulation at $T_{24}$ and $T_{48}$ . It then declined at $T_{72}$ of the synchronization period. There were interactions ( P<0.01 ) between the treatment and synchronization periods ( $T_{0}$ – $T_{72}$ h). The higher values of the estradiol 17- β concentration were recorded in G4 and G3 (67.30 ± 2.69 and 61.80 ± 2.69 pg mL ${}^{-1}$ , respectively) at $T_{48}$ , while the lower values were recorded in G1 at the time (0–72 h) of the synchronization period together with the overall mean for this group (Table 4).

As shown in Table 5, there was a significant effect ( P<0.05 ) on the overall mean concentrations of progesterone (P ${}_{4}$ ) among the different groups during early pregnancy. The highest concentration was observed in G4 (10.54 ± 0.45 ng mL ${}^{-1}$ ), while the other groups, G1, G2, and G3, had concentrations of 3.44 ± 0.45, 5.40 ± 0.45, and 3.50 ± 0.45 ng mL ${}^{-1}$ , respectively. There were significant interactions ( P<0.05 ) between the treatment and the early pregnancy period. The results of this study revealed that ewes treated with the eCG hormone only (G2) or in combination with CSFA (G4) had higher progesterone concentrations compared to ewes treated with CSFA alone (G3) or untreated ewes (control group G1).

**Table 5:** Variations of blood plasma progesterone concentrations (ng mL ${}^{-1}$ ) during two stages (luteal stage and early pregnancy stage) in the experimental groups.

Experimental group	Progesterone profile			± SE	Overall	± SE
	Luteal stage	Early pregnancy				
	8 d	18 d	30 d			
G1	2.39 ${}^{d}$	2.39 ${}^{d}$	5.53 ${}^{\mathrm{bc}}$	0.78	3.44 ${}^{C}$	0.45
G2	4.11 ${}^{\mathrm{cd}}$	5.33 ${}^{\mathrm{bc}}$	6.77 ${}^{b}$		5.40 ${}^{B}$	
G3	4.01 ${}^{\mathrm{cd}}$	3.33 ${}^{\mathrm{cd}}$	3.16 ${}^{\mathrm{cd}}$		3.50 ${}^{C}$	
G4	9.84 ${}^{a}$	11.60 ${}^{a}$	10.19 ${}^{a}$		10.54 ${}^{A}$	
Overall	5.09	5.66	6.41	0.38		

SE: standard error. ${}^{a,d}$ : means in the same rows not followed by the same superscript letter are significantly different ( p<0.01 ). ${}^{A,B}$ : means in the same column not followed by the same superscript letter are significantly different ( p<0.05 ).

**Table 6:** Reproductive performance traits of Barki ewes supplemented with CSFA and/or superovulated with hormonal treatment.

Groups	Traits								
	Sex	Total live-	Sex ratio	Conception	Lambing	Prolificacy	Single	Twinning	Quad
		born lambs	(%)	rate of 30 d (%)	rate (%)	rate (%)	rate (%)	rate (%)	rate (%)
G1	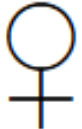	2	40	66.66 (6/9)	55.55 (5/9)	100 (5/5)	100 (5/5)	0 (0/5)	0 (0/5)
	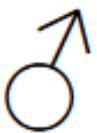	3	60						
	Total	5	100.0						
G2	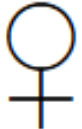	5	45.45	90 (9/10)	90 (9/10)	122.22 (9/11)	77.77 (7/9)	22.22 (2/9)	0 (0/9)
	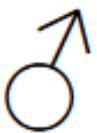	6	54.54						
	Total	11	100.0						
G3	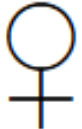	6	60	80 (8/10)	80 (8/10)	125 (10/8	75 (6/8)	25 (2/8)	0 (0/8)
	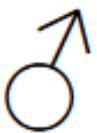	4	40						
	Total	10	100.0						
G4	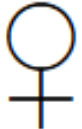	8	50	100 (10/10)	100 (10/10)	160 (16/10)	60 (6/10)	30 (3/10)	10 (1/10)
	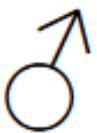	8	50						
	Total	16	100						

Table 6 presents the total number of live-born lambs, sex ratio, conception rate, lambing rate, prolificacy rate, single rate, twinning rate, and quadruplet rate of Barki ewes. The results showed that treatment with eCG and/or CSFA in groups G2, G3, and G4 improved the number of total live-born lambs (11, 10, and 16, respectively) compared to the control group G1 (5 live-born lambs).

The treatment of ewes with eCG and/or CSFA also improved conception rates after 30 d of mating and lambing rates in groups G2, G3, and G4 (90 %, 80 %, and 100 %, respectively) compared to the conception rate in the control group G1 (66.7 %) and the lambing rate in group G1 (55.6 %). A similar trend of improvement was observed in prolificacy rates in groups G2, G3, and G4 (122.2 %, 125.0 %, and 160 %, respectively) compared to the control group G1 (100 %). Furthermore, the treatment resulted in a significant improvement in twinning rates in groups G2, G3, and G4 (22.2 %, 25.0 %, and 30.0 %, respectively) compared to the control group G1 (0.0 %). Overall, there were no statistically significant differences ( P>0.05 ) in the total number of live-born lambs, sex ratio, conception rate, lambing rate, prolificacy rate, single rate, twinning rate, and quadruplet rate among the treatment groups.

Although there were no significant differences between the control and treated groups, the ewes treated with CSFA and/or eCG hormones tended to enhance the reproductive traits of Barki ewes. These treatments increased the total live offspring, conception rate (at day 30 of mating), lambing rate, prolificacy rate, and twinning rate and decreased single births. This effect was clearer in the combination group (G4), which had higher values in all the reproductive traits compared to the other treated groups (G2 and G3) and the control group (G1). It is interesting to note that, in the present study, only the fourth group (G4; CSFA-fed ewes plus eCG injection) had quadruplet birth rates (10 %; Table 6).

## 4 Discussion

The results of this study demonstrated a positive effect of CSFA, alone or in combination with an estrous synchronization protocol, on the ovarian activity of Barki ewes, as indicated by the number and sizes of follicles. Similar findings have been observed in previous studies, such as El-Shahat and Abo-El Maaty (2010), who concluded that the addition of calcium salts of long-chain fatty acids to the diet significantly improved the number and sizes of ovarian preovulatory follicles in Rahmani ewes. Additionally, De-Fries et al. (1998) reported that dairy cows fed supplemental fat had larger and more preovulatory follicles compared to non-fed cows. The energy content of diets can influence follicular development through direct actions of nutrients on the ovarian microenvironment (Guo et al., 2017; Grazul-Bilska et al., 2018) and the development of gonadotropin receptors (Ying et al., 2011). Dietary fat may enhance follicular development through metabolites and metabolic hormones that influence GnRH (gonadotropin-releasing hormone) secretion (De-Fries et al., 1998). Furthermore, nutritional supplementation in combination with estrous synchronization has been shown to enhance the number of larger follicles (Nogueira et al., 2016). Studies on beef and dairy cows by Hightshoe et al. (1991) and Lammoglia et al. (1997) found that a high-fat diet increased the number of medium-sized follicles, from which the preovulatory follicles were selected. Similarly, Hashem and El-Zarkouny (2017) demonstrated that rumen-protected fat supplementation modulated the metabolic profile of ewes and provided specific fatty acids important for the quality of the ovulatory follicle and ovulation activity. Regarding the hormonal (PGF + eCG) treatment (alone or in combination with protected fat) effect on ovarian follicles, our results revealed that the injection with 600 IU of eCG after receiving double doses of prostaglandin had a positive effect on ovarian follicles, as indicated by the large-sized follicles ( ≥5 mm), which were significantly higher in ewes treated with eCG (G2 and G4) compared to the control group (G1). The enhancement in the overall mean diameter of large follicles may be due to the eCG effect. Actually, eCG has bi-functional possible FSH (follicle-stimulating hormone)- or LH (luteinizing hormone)-like functions that can increase follicular development (Rebollar et al., 2006; Lorenzo et al., 2014), supports follicular growth (Ainsworth and Shrestha, 1985), and enhances the recruitment of small follicles (Noel et al., 1994).

The current data observed that the ewes treated with protected fat and/or eCG had significantly higher plasma estradiol concentrations during the synchronized period ( $T_{0}$ – $T_{72}$ h) and the overall mean values of this period compared to untreated ewes. This effect was more obvious in the combination group (G4). It seems that the increase in concentrations of estradiol 17- β (E ${}_{2}$ ) may be associated with the maturation of follicles ( ≥5 mm) in the ovaries of ewes of the treated groups (G2, G3, and G4). In the present study, treatment with eCG and/or CSFA enhanced the diameters of large follicles as shown in Table 3. These large follicles are considered the main source of the estradiol (Keyes and Nalbandov, 1967; Niswender et al., 2000) responsible for the manifestation of estrus and its intensity. It has been reported that the higher doses of eCG (400–600 IU) would presumably lead to more estrogen production (Moakhar et al., 2012; D'Souza, 2013) from the largest follicle, which is the principal source of estradiol (Munoz-Gutierrez et al., 2002). On the same topic, Hashida et al. (2013) showed that eCG injections managed to increase plasma estradiol 17- β concentrations in synchronized ewes. Similarly, Barrett et al. (2004) and Habibizad et al. (2015) found that the mean serum estradiol concentrations for the pre- and early post-ovulatory periods were higher in eCG-treated ewes compared to the control group. Furthermore, ewes treated with eCG had the greatest ( P<0.05 ) concentration of estradiol produced from large follicles of ewes with two ovulations (Habibizad et al., 2015).

The increase in the serum concentration of estrogen can be attributed to the increase in lipid metabolites, as cholesterol is the precursor of all steroids and increased available substrate may increase steroid synthesis and improve the energy balance by supplemented fat (Abayasekara and Wathes, 1999). Unfortunately, the concentrations of cholesterol were not determined in this study to confirm whether this was in fact the case. However, more recent results obtained by Abd El-Hamid et al. (2016) showed that provision of a supplement like protected fats prior to, during, and following mating increased serum lipid metabolites, including cholesterol during the treatment period. Also, it is interesting to note that the CSFA source (Magnapac^®^) in our study contained palmitic acid, oleic acid, and linoleic acid. Actually, it is known that an increased availability of precursor fatty acids in diets is coupled with increased steroid synthesis. Robinson et al. (2002) found that dairy cows supplemented with a fat containing either high concentrations of oleic acid or high concentrations of linoleic acid had higher estradiol concentrations in plasma than the control group. The obtained results in the present study showed that the estradiol 17- β concentrations had decreased at 72 h of the synchronization period (Table 4). This may be due to the ovulation or may be the estradiol decrease correlated with LH surge. Regrettably, the ovulation time and LH peak were not determined in this study to confirm whether this was in fact the case, but some previous studies can support the hypothesis. Komar et al. (2001) suggested that the capacity of the preovulatory follicle to secrete estradiol is significantly impaired by about halfway through the peri-ovulatory interval. Moreover, the estradiol concentration in the fluid of preovulatory follicles decreases about 6 h after the preovulatory peak of LH in cows (Dieleman et al., 1983).

The increase in progesterone concentration after these treatments may be due to the positive effects of eCG or CSFA on ovulation rate, leading to an increase in the number of CLs as well as the number of embryos. This effect was more pronounced in G4 (CSFA + eCG treated ewes), which had higher rates of twinning and quadruplet births, as shown in Table 6. These findings suggest that treatment with eCG and/or dietary-protected fat improves ovulation and CL formation.

Previous studies have reported a direct relationship between the number of CL and the amount of progesterone secreted in rats (Elbaum et al., 1975). The number of corpora lutea is closely associated with the number of embryos in the uterus (Deanesly, 1966). El-Shahat and Abo-Elmaaty (2010) found that supplementation of CSFA improves the ovarian response of ewes and increases both ovulation rate and serum progesterone levels. These results are consistent with the study by Kuran et al. (1999) that reported enhanced luteal progesterone synthesis in ewes given dietary supplements of palmitic acid, stearic acid, and oleic acid in the form of rumen-protected CSFA. Feeding CSFA diets was also found to increase progesterone concentrations in dairy cows and delay CL regression (Garcia-Bojalil et al., 1998; Moallem et al., 1999). Additionally, Hightshoe et al. (1991) found that feeding supplemental fat to beef cows improved progesterone concentrations during the luteal phase of the first postpartum estrous cycle. Progesterone is essential for pregnancy maintenance, and one of the important functions of the blastocyst is to counteract the uterine luteolytic mechanism. Progesterone plays a crucial role in preparing the uterus for embryo development and implantation. Therefore, increasing progesterone levels during early pregnancy reduces embryonic losses and increases pregnancy rate and fertility (Ataman et al., 2013).

Interestingly, a marked increase in ewes giving birth to female offspring was observed in the CSFA-treated groups as compared to the control group. A similar observation was reported by Morsy et al. (2008) in Barki ewes receiving two doses of CSFA where higher percentages of females were born compared to the control group. Published data concerning the effect of food supplements on sex ratios are controversial and mostly related to diet composition during mating and the fertilization period (Flint et al., 1997; Landete-Castillejos et al., 2004).

Gulliver et al. (2013) reported that further research is required to determine whether the observed increase in the proportion (58.2 % vs. 43.5 %) of female born lambs of the Merino × Border Leicester ewes fed high PUFA diets prior to and following conception was due to specific alterations in the n -6 fatty acids or other differences in the diet. However, alteration of the unsaturated fatty acid profile in the diet, being rich in oleic acid during mating and the fertilization period, may explain the skewed lamb sex ratio to female rather than male lambs (Morsy et al., 2008; Abd El-Hamid et al., 2016).

The results concerning conception, lambing, and prolificacy rates in our study were in agreement with those of Hegazy et al. (1999), who reported that lambing, pregnancy, and prolificacy rates were improved in Barki ewes fed CSFA. Moreover, Safdar et al. (2013) found that the synchronized ewes (CIDR – controlled internal drug release – and PMSG – pregnant mare serum gonadotropin) supplemented with CSFA from two sources (sunflower oil and flaxseed oil) had higher values in total offspring, pregnancy, and prolificacy rates as compared to the untreated ewes. Adding CSFA to the diet increased serum concentrations of P ${}_{4}$ in bovine females (Hightshoe et al., 1991). High concentrations of P ${}_{4}$ before and after breeding are associated with enhanced fertility (Folman et al., 1973) and embryonic survival (Mann et al., 2006). Furthermore, the current results were in agreement with Abd El-Hamid et al. (2016), who concluded that including CSFA in a flushing diet for Barki ewes resulted in improved twinning, prolificacy rates, and female–male lamb sex ratios. The CSFA source (Magnapac^®^) in this study contained palmitic acid, oleic acid, and linoleic acid (as shown in Table 2). Supplementation of protected fat turned out to enhance conception rates, and there were clear trends for improving lambing and the prolificacy rate. This can be explained on the basis of several mechanisms; supplementation of rumen bypass fatty acids originating from palm oil to ewes increases the number of high-quality oocytes and thus the embryo survival rate because both oleic acid and palmitic acid are the major fatty acids contributing to a ovine zona pellucid structure (McEvoy et al., 2000; Zeron et al., 2002; Ashworth et al., 2010). Additionally, supplementation of CSFA to the given diet may have provided Ca ${}^{++}$ ions that played an important role in semen capacitation in mated females, resulting in improved conception and lambing rates (Visconti et al., 2002). Regarding the positive effects of eCG (irrespective of CSFA treatments) on the reproductive activity of Barki ewes in our study, it has been documented that eCG increased the ovulation rate, multiple births, and the number of lambs born per ewe lambed as reported by Akoz et al. (2006).

## 5 Conclusion

The present study suggests that dietary supplementation of CSFA (Magnapac^®^) in Barki ewes prior to mating is effective in maintaining reproductive performance under semiarid conditions. This improvement is evident through increased levels of reproductive hormonal profiles, enhanced numbers and sizes of ovarian preovulatory follicles, and improved rates of conception, lambing, prolificacy, and twinning rates. In fact, the effects of protected fats are most pronounced when combined with hormonal treatments such as PGF ${}_{2}\alpha$ + eCG.

## Data Availability

The authors confirm that the data supporting the findings of this study are available within the article.
